# Results expression for tests used to measure the anticoagulant effect of new oral anticoagulants

**DOI:** 10.1186/1477-9560-11-9

**Published:** 2013-06-28

**Authors:** Armando Tripodi

**Affiliations:** 1 Angelo Bianchi Bonomi Hemophilia and Thrombosis Center, Department of Clinical Sciences and Community Health, Università degli Studi di Milano and IRCCS Cà Granda Maggiore Hospital Foundation, Via Pace 9, Milano, 20122, Italy

## Abstract

Results of clotting tests used to measure the effect of old and new antithrombotic drugs can be expressed in different ways and this is considered as one of the sources of variability to explain the differences of results obtained for the same patient plasma when tested in different laboratories. This is particularly important for patient on vitamin K antagonists and led to the development of the international normalized ratio system of results reporting in this setting. Although standardization of results expression for the tests meant to measure the anticoagulant effect of new oral anticoagulants (NOA) is presently not perceived as an issue, it may become crucially important at the time when test-specific cut off values will be available to help assessing the risk of bleeding in individual patients who are on over-dosage. Effort should therefore be made to harmonize as much as possible results obtained in different laboratories using the same method, but different reagents. This article is aimed at discussing different options of results reporting of tests for NOA and their merits/pitfalls.

## Introduction

Although clinical trials for the new oral anticoagulants (NOA) [1] demonstrated that they do not require dose-adjustment by means of laboratory testing, the measurement of their anticoagulant effect may be useful in many circumstances including preoperative screening; patients admitted to the emergency department because of adverse events (hemorrhage or thrombosis); patients with chronic renal insufficiency; patients taking other drugs that are known or suspected to interact with the NOA. In all these circumstances few tests may be required and according to recent recommendations they are the prothrombin time (PT) test or the measurement of the anti-factor Xa activity for rivaroxaban and the dilute thrombin time (dTT) or the ecarin clotting time (ECT) for dabigatran [2,3]. The results of these tests may be expressed in different ways and no specific recommendations have hitherto been issued. This article is aimed at reviewing the most important results expression and their relevance for the laboratory measurement of the anticoagulant effect of NOA.

## Prothrombin time and anti-factor Xa for rivaroxaban

### Prothrombin time

Results for PT are commonly reported as (i) clotting times (seconds); (ii) ratio of patient-to-normal clotting times [i.e., PT-ratio = (PT_patient_/PT_normal_)] or as (iii) international normalized ratio (INR) [i.e., INR = (PT_patient_/PT_normal_)^ISI^] where the ISI is the international sensitivity index of the thromboplastin used for testing and represents its responsiveness to the PT prolongation mediated by vitamin K antagonists (VKA) relatively to the World Health Organization (WHO) international standard for thromboplastin [4]. The INR has been devised to harmonize PT results across thromboplastins, but only for patients on VKA. Hence, any other application should be validated before use to see whether it is able to minimize between-thromboplastin results. For example, it was recently shown that the regular INR is unable to minimize PT results for patients with chronic liver disease [5]. However, the same authors showed that the system could be reliably used also in this setting provided that modification in the determination of the ISI was introduced [5]. Similarly, other authors provided evidence that appropriate modifications of the ISI makes the INR scale suitable to minimize the between-thromboplastin differences of PT results for patients with disseminated intravascular coagulation [6] or to minimize between-reagent differences for coagulation testing to detect lupus anticoagulants [7]. All the above observations can be taken as evidence that the INR is a system suitable to minimize between-reagent variability of coagulation test results provided that specific sensitivity indexes relatively to common standards are calculated for different clinical conditions.

Recently, it has been shown that PT results, expressed as clotting times (seconds) or PT-ratio, for patients on rivaroxaban vary according to the thromboplastin used for testing [8]. More recently, this variability was quantified by measuring the PT for normal plasmas spiked with known amounts of rivaroxaban to mimic plasmas from patients on treatment [9]. The average PT-ratios (patient-to-normal) for three plasmas at rivaroxaban concentrations of 100, 300 or 700 ng mL^−1^ were 1.27, 1.76 or 2.67, respectively [9]. The between-thromboplastin variability calculated as the coefficient of variation (CV) of results obtained with six commercial thromboplastins, testing each of the above plasmas, amounted to 5.5%, 12.1% and 18.1% (average value, 14%) [9].The above mentioned variability was dramatically increased when results were expressed as INR with CV values of 10.4%, 24.6%, or 39.0% (average value, 29.6%) [9]. These results tell us that the between-thromboplastin variability of the PT-ratio for plasmas containing rivaroxaban is relatively high and that the regular INR is unable to minimize such variability as it occurs for patient on VKA. Hence, the regular INR cannot be used to express results for patients on rivaroxaban.

**Figure 1:**
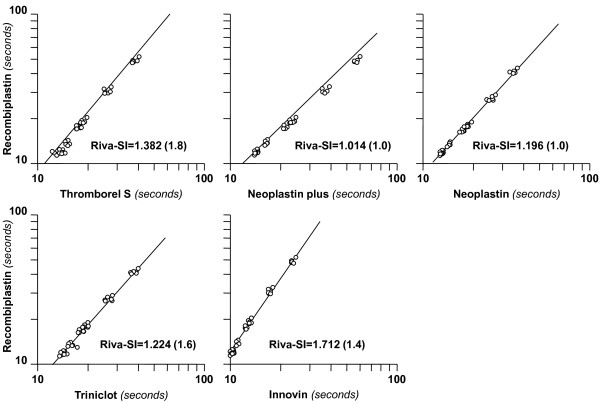
**Calibration of thromboplastins to determine the Rivaroxaban sensitivity index (here referred as *Riva-SI*).** Aliquots of a pooled normal plasma were spiked with increasing amounts of rivaroxaban. Plasmas were then tested with five commercial thromboplastins and with a common thromboplastin (referred as common standard). Prothrombin time (PT) results (seconds) were then plotted on a double-log scale (common standard on the vertical axis) and the best-fit line was drawn. The slope of the line, estimated by the orthogonal regression analysis is the *Riva-SI* and can be taken as a measure of the responsiveness of the rivaroxaban-induced PT prolongation of the five thromboplastins relatively to the common standard. The *Riva-SI* can be used to convert PT results (seconds) into rivaroxaban standardized PT ratio (here referred as *Riva-PT-ratio*) according to the following equation: *Riva-PT-ratio = [PT*_*patient*_*/PT*_*normal*_*]*^*Riva-SI*^. This system of standardization was effective in minimizing the between-thromboplastin variability of the PT for rivaroxaban-spiked plasmas (see text more details). The *Riva-SI* relatively to the common standard is reported on each graph. By definition the higher the *Riva-SI* the lower the responsiveness of the thromboplastin to the effect induced by rivaroxaban. The numbers in brackets represent the coefficient of variation of the slope estimation. The *Riva-SI* for the common standard has been arbitrarily set at 1.00. Results did not change appreciably when Neoplastin Plus was used as the common standard [9].

Subsequent studies showed that there may be an additional expression of PT results suited to be applied in patients on rivaroxaban [9]. It was hypothesized that the responsiveness of the clotting time prolongation of working thromboplastins induced by rivaroxaban could be determined by testing plasmas added with increasing amounts of rivaroxaban with the working and standard thromboplastins. If the paired PTs (seconds), plotted on a double-log scale, are linearly related, the slope of the orthogonal regression line can be taken as an index of the responsiveness of the working thromboplastin relatively to the international standard (Figure 1). By definition, a slope value equal to the unity denotes that the working thromboplastin possesses the same responsiveness to rivaroxaban as the standard thromboplastin. Likewise, the greater the slope the lower the responsiveness. This model of calibration that has been described in detail [9], adopted the same statistical procedure recommended by WHO for the determination of the ISI of thromboplastins used for patients on VKA [4]. The numerical value of the slope, provisionally called rivaroxaban sensitivity index (*Riva-SI*), can then be used to convert results of the PT (seconds) into a new scale, called rivaroxaban standardized ratio (*Riva-PT-ratio*), according to the equation:

$$\mathrm{Riva}-\mathrm{PT}-\mathrm{ratio}={\left({\mathrm{PT}}_{\mathrm{patient}}/{\mathrm{PT}}_{\mathrm{normal}}\right)}^{\mathrm{Riva}-\mathrm{SI}}$$

This system of standardization proved feasible [9] for the following reasons. (i) Six thromboplastins testing plasmas spiked with rivaroxaban yielded results that were linearly related to those of a common thromboplastin standard; (ii) the data points could be represented by a best-fit orthogonal regression line and (iii) the slope of the line could be estimated with a high degree of confidence with CV value (that is a measure of the calibration precision) less than the required 3% [4] (see Figure 1). The responsiveness to rivaroxaban was the highest for Neoplastin Plus (Stago, Asnieres, France) and Recombiplastin (Instrumentation Laboratory, Orangeburg, NY) (i.e., *Riva-SI* close to unity) and the lowest for Innovin (Siemens Health Care Diagnostics, Marburg, Germany) (*Riva-SI* = 1.712) (see Figure 1). Interestingly, the responsiveness to rivaroxaban does not depend on the species of the thromboplastin as shown by the evidence that two human recombinant thromboplastins such as Recombiplastin and Innovin display similar responsiveness to VKA, but completely different responsiveness to rivaroxaban (see Figure 1).

In the second step of the study, the above system of standardization was validated by testing three rivaroxaban-spiked plasmas (that were different from those used for the determination of the *Riva-SI*) [9]. The overall between-thromboplastin variability of the PT results that was on average 14.1% or 29.6% when results were expressed as *PT-ratio* or *INR* was reduced to a mere 2.1% when results were expressed as *Riva-PT-ratio*[9]. These results and conclusions were recently confirmed by Harenberg et al. [10].

The *Riva-PT-ratio* although feasible should be further investigated to address practical issues that have been previously discussed in detail [9]. For instance, in the model so far proposed normal plasmas spiked with increased amounts of rivaroxaban have been used to determine the *Riva-SI* for the working thromboplastins. These plasmas although representative do not necessarily mirror the real situation of plasmas from patients treated with rivaroxaban. This issue had already been considered for the calibration of the INR in patients on VKA and guidelines have been issued on preparation, certification and use of certified plasmas for ISI calibration and INR determination [11]. Further work is, therefore, needed to give specific details on the preparation, certification, validation and use of rivaroxaban-spiked plasmas. In this respect, some work has already been carried out [12], but much remains to be done. Upon further investigation and validation the *Riva-PT-ratio* could be implemented in clinical laboratories. In practice, PT results for patients on rivaroxaban could be converted into *Riva-PT-ratio*s by using the above equation. The parameters needed are the PT_normal_ and the *Riva-SI* value for the working thromboplastin. The first may be calculated as the geometric mean PT value of 20 or more healthy subjects or as the clotting time of the pooled normal plasma [4]. The second should be determined by the manufacturers by testing with their thromboplastins and with the international standard for thromboplastin (available from WHO) a set of rivaroxaban-spiked plasmas according to the described protocol [9]. This system of reporting does not require the use of calibration plasmas in the local laboratories and would reduce the cost incurred for patient management.

It was proposed that the results of the PT test for patients on rivaroxaban can also be reported as rivaroxaban-equivalent concentrations [12]. This may be achieved by testing in local laboratories the PT for a set of plasmas spiked with known and increasing amounts of rivaroxaban. The PT value (seconds) can then be plotted on a linear scale versus the rivaroxaban concentrations and used to construct local calibration curves. Patients clotting times can be interpolated from these calibration curves and the results converted directly into rivaroxaban-equivalent concentrations. The validity of this system of reporting rests on the assumption that the interaction between rivaroxaban and plasma does not vary in individual patients, an assumption that, however, cannot be taken for granted. For instance, it is reasonable to assume that calibration curves prepared by different normal plasmas spiked with the same amounts of rivaroxaban may yield different slope and intercept. Hence, the drug concentrations derived thereafter for the same patient could be different, depending on the curve used for the interpolation. Furthermore, it should be realized that patients to be treated with rivaroxaban may have variable baseline PT values and often different from the value of the normal plasma used for spiking. This variability may have unpredictable effect on results expression as drug concentration-equivalent.

In conclusion, based on the present limited experience, I believe that PT results for patients on rivaroxaban can be expressed as *PT-ratio*, possibly as *Riva-PT-ratio* (if this system will be implemented), but not as INR as this expression dramatically magnify the between-thromboplastin variability.

### Anti-factor Xa

The assay is based on the capacity of rivaroxaban to quench factor Xa activity added in excess to the test plasma. The residual factor Xa activity that is inversely proportional to the rivaroxaban activity is measured by means of a specific synthetic substrate. The results are expressed primarily in terms of optical density and subsequently converted into rivaroxaban concentrations by interpolation from a calibration curve prepared locally by testing plasmas spiked with increasing concentrations of rivaroxaban [13]. Although this expression of results is the obvious solution, it should be realized that it might not be an adequate system of standardization as shown by the rather large inter-laboratory variability that was observed in a multicenter survey of anti-factor Xa methods for rivaroxaban [14]. In the survey, 21 laboratories were asked to measure rivaroxaban concentrations in the same plasma using their local reagents. The resultant calculated inter-laboratory CV value amounted to 25%, despite the fact that local reagents for anti-factor Xa determination were calibrated with the same set of calibration plasmas [14]. On the other hand, the lesson learnt from the attempt to standardize the anti-factor Xa assay for low molecular weight heparins [15] tells us that even in the setting of rivaroxaban, standardization across reagents is far reaching. In practical terms this means that any cut-off value, which will eventually be determined on the concentration of drug obtained with a specific anti-factor Xa method, cannot be easily generalized to other reagents.

## Dilute thrombin and ecarin clotting times for patients on dabigatran

The dilute thrombin and ecarin clotting times (dTT and ECT) are tests that can be used to measure the anticoagulant effect of dabigatran. The activated partial thromboplastin time (APTT) would also be responsive to dabigatran, but dose–response is not linear. The PT is rather unresponsive to dabigatran.

dTT is based on recording the clotting time of a mixture of test plasma and optimal amounts of thrombin. Dabigatran quenches the activity of thrombin and therefore the clotting time prolongation of dTT is proportional to the inhibitory activity of the drug.

ECT is based on the property of the snake venom *ecarin* to convert prothrombin into meizothrombin, which can in turn be measured by recording the prolongation of the plasma clotting time or by a synthetic chromogenic substrate [16]. Dabigatran quenches meizothrombin and therefore the clotting time or the chromogenic activity will be proportional to the activity of dabigatran.

Results for dTT or ECT are usually expressed as clotting time (seconds). They can also be expressed as ratio (patient-to-normal). Both dTT and ECT proved fairly responsive to dabigatran and commercial assays are available. The clotting times are approximately 2.0- or 3.0-times longer than the baseline value for dTT or ECT, respectively, when the concentration of dabigatran is 200 ng/mL [17]. The latter being the plasma concentration that can be found in patients taking 150 mg dabigatran twice daily. It is, therefore, reasonable to assume that the prolongations of the clotting times for both tests will be even higher in patients on over-dosage, that is the situation when testing will probably be most useful. Cut off values for both tests could be established in clinical studies to see whether they may be useful to identify patients at increasing risk of hemorrhage. However, for these cut off to be used *“universally”* (i.e., regardless of the reagent used for testing) results obtained in different laboratories should be comparable. Although the formulation of dTT and ECT is very simple, it is anticipated that results (even if they are expressed as ratio) will vary between laboratories. Different types (human or bovine) and concentrations of thrombin will probably be used by different commercial kits, which will ultimately make results to vary in different labs. Standardization of ECT would in principle be easier since the source of the snake venom (*ecarin*) that is used to activate prothrombin comes from the same source, but application of this test to different clot detection systems or chromogenic substrates may introduce some degree of variability.

As mentioned above for rivaroxaban, results of tests for dabigatran could also be expressed as dabigatran-equivalent concentrations [18]. This requires provision of sets of calibration plasmas (that are commercially available) prepared by spiking a normal plasma with increasing concentrations of the drug. Calibration plasmas could be tested with local reagents and equipment and the results used to construct calibration curves. Patients clotting time or chromogenic activity can in turn be converted by means of these calibration curves into dabigatran-equivalent concentrations. Presumably this system of reporting will experience the same problems of standardization discussed above for rivaroxaban.

## Conclusions

Results of clotting tests can be expressed in different ways and this was always considered as one of the most important source of variability to explain the differences of results obtained for the same patient plasma when tested in different laboratories. This state of affairs, was deemed crucial for patient on VKA and led to the development of the INR system of results reporting.

Although standardization of results expression for the tests used to measure the anticoagulant effect of NOA is presently not perceived as an issue, it may become crucially important at the time when test-specific cut off values will be available to help assessing the risk of bleeding in individual patients who are on over-dosage. Effort should, therefore, be made to harmonize as much as possible results obtained in different laboratories using the same method, but different reagents.

Whether results of coagulation tests should eventually be expressed as (standardized) ratio (patient-to-normal) or as drug concentration-equivalent, interpolated from a dose–response curve constructed by testing normal plasmas added with increasing amounts of any of the NOA, is still a matter of debate and should be explored by appropriate clinical studies.

## Competing interests

The author declares that he has no competing interests.
